# Crystallization and preliminary X-ray characterization of the tetrapyrrole-biosynthetic enzyme porphobilinogen deaminase from *Bacillus megaterium*

**DOI:** 10.1107/S1744309113018526

**Published:** 2013-07-27

**Authors:** N. Azim, E. Deery, M. J. Warren, P. Erskine, J. B. Cooper, S. P. Wood, M. Akhtar

**Affiliations:** a School of Biological Sciences, University of Punjab, New Campus, Lahore 54590, Pakistan; b School of Biosciences, University of Kent, Stacey Building, Canterbury, Kent CT2 7NJ, England; c Laboratory of Protein Crystallography, Centre for Amyloidosis and Acute Phase Proteins, UCL Division of Medicine (Royal Free Campus), Rowland Hill Street, London NW3 2PF, England

**Keywords:** tetrapyrrole biosynthesis, porphobilinogen deaminase, *Bacillus megaterium*, dipyrromethane cofactor

## Abstract

The enzyme porphobilinogen deaminase (PBGD; hydroxymethylbilane synthase; EC 2.5.1.61) catalyses an early step of the tetrapyrrole-biosynthesis pathway in which four molecules of the monopyrrole porphobilinogen are condensed to form a linear tetrapyrrole. The enzyme possesses a dipyrromethane cofactor which is covalently linked by a thioether bridge to an invariant cysteine residue. Expression in *Escherichia coli* of a His-tagged form of *Bacillus megaterium* PBGD permitted the crystallization and preliminary X-ray analysis of the enzyme from this species at high resolution.

## Abstract 2

The enzyme porphobilinogen deaminase (PBGD; hydroxymethylbilane synthase; EC 2.5.1.61) catalyses a key early step in the biosynthesis of tetrapyrroles in which four molecules of the monopyrrole porphobilinogen are condensed to form a linear tetrapyrrole. PBGD from *B. megaterium* was expressed and the enzyme was crystallized in a form which diffracts synchrotron radiation to high resolution.

## 1. Introduction   

**Figure 1:**
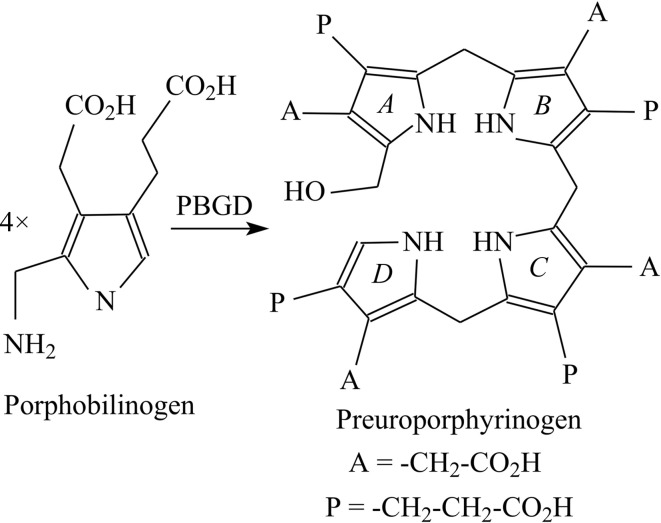
The reaction catalysed by porphobilinogen deaminase. Four molecules of the pyrrole porphobilinogen are condensed to form the linear tetrapyrrole preuro­porphyrinogen (hydroxymethylbilane). The acetic and propionic acid side chains of each pyrrole are abbreviated A and P, respectively, and the four rings of the tetrapyrrole product are indicated in italics as *A*, *B*, *C* and *D*.

**Figure 2:**
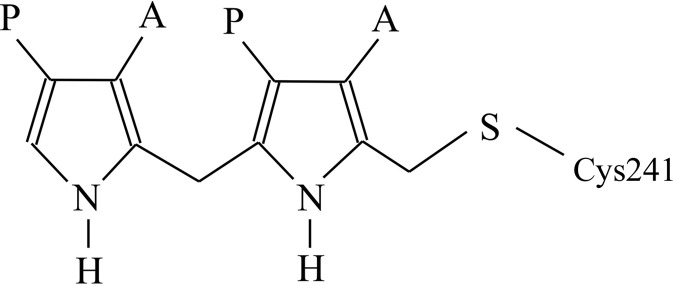
The dipyrromethane cofactor of porphobilinogen deaminase is covalently attached to the enzyme by a thioether bond to a cysteine residue. Four substrate pyrroles are added linearly to the cofactor and, finally, hydrolysis of the linkage between the substrate and the cofactor releases the tetrapyrrole product hydroxymethylbilane.

The enzyme porphobilinogen deaminase (PBGD), which is also known as hydroxymethylbilane synthase (EC 2.5.1.61), catalyses an early step of the tetrapyrrole-biosynthesis pathway in which four molecules of the monopyrrole porphobilinogen are condensed to form a linear tetrapyrrole, preuroporphyrinogen or hydroxymethyl­bilane (Fig. 1 ▶; Jordan, 1991 ▶). Isotopic labelling and single-turnover studies showed that the pyrrole-forming ring *A* (Fig. 1 ▶) is the first to bind to the enzyme, followed by rings *B*, *C* and finally *D*. PBGD is a monomeric protein with a molecular weight in the range 34–44 kDa depending on the species (Jordan, 1991 ▶). The enzyme possesses a dipyrromethane cofactor (Fig. 2 ▶) which is covalently bound to the enzyme by a thioether linkage involving an invariant cysteine residue (Jordan & Warren, 1987 ▶). The cofactor acts as a primer to which four porphobilinogen molecules are attached sequentially prior to cleavage of the link between the cofactor and the first substrate molecule on completion of the reaction. Thus, the cofactor remains covalently attached to the enzyme when the product of the reaction is released.

The X-ray structure of the *Arabidopsis thaliana* enzyme has recently been solved (PDB entry 4htg; Roberts *et al.*, 2013 ▶); prior to this, structures were available for *Escherichia coli* PBGD [PDB entries 1pda (Louie *et al.*, 1992 ▶, 1996 ▶), 1gtk (Helliwell *et al.*, 2003 ▶), 1ah5 (Hädener *et al.*, 1999 ▶), 1ypn (Helliwell *et al.*, 1998 ▶) and 2ypn (Nieh *et al.*, 1999 ▶)] and the human enzyme (PDB entries 3eq1 and 3ecr; Gill *et al.*, 2009 ▶; Song *et al.*, 2009 ▶). The polypeptide is folded into three domains (1–3), each of approximately the same size. The general architecture of domains 1 and 2 shows a strong resemblance to a number of periplasmic binding proteins. The dipyrromethane cofactor is attached to a loop on domain 3 and is positioned at the mouth of a deep active-site cleft formed between domains 1 and 2.

The Gram-positive bacterium *Bacillus megaterium* is of great industrial interest since it has many commercial applications in the biotechnological production of numerous substances, including the tetrapyrrole vitamin B_12_. Here, we report the expression and crystallization of PBGD from *B. megaterium* in a form that diffracts synchrotron radiation to very high resolution.

## 2. Expression and purification   

The *B. megaterium* PBGD gene was cloned into the *Nde*I and *Bam*HI restriction sites of the expression vector pET14b using standard methods and was transformed into Rosetta (DE3) *E. coli* cells (Novagen). Expression was undertaken using 500 ml cultures, which were grown overnight at 310 K following induction with 0.2 m*M* isopropyl β-d-1-thiogalactopyranoside (IPTG) at mid-log phase. The cultures were then centrifuged at 5000 rev min^−1^ for 15 min with a Beckman Coulter Avanti J-26 XP ultracentrifuge using a JLA-8.1000 rotor to obtain the cell pellet. The 3.75 g pellet obtained from a 1 l culture was then resuspended in 25 ml 50 m*M* Tris–HCl buffer pH 8 and sonicated on ice using an MSI Soniprep 150 instrument. The cell lysate was centrifuged at 12 000 rev min^−1^ using a Beckman JA-25.50 rotor to separate the cell debris from the supernatant. The clear supernatant was then loaded onto a pre-equilibrated HisTrap HP (GE Healthcare) 1 ml column, allowing the His-tagged PBGD to bind prior to washing the column with binding buffer in order to remove impurities. The enzyme was eluted with a buffer containing 500 m*M* imidazole and the polyhistidine tag was removed by the addition of thrombin (one unit per milligram of purified protein) followed by overnight incubation at room temperature. The cleaved tag and thrombin were then removed by passing the previously dialysed protein through a HisTrap HP 1 ml column again followed by a HiTrap Benzamidine FF 1 ml column (GE Healthcare). The final yield of purified *B. megaterium* PBGD was approximately 10–12 mg per litre of cell culture. Curiously, the cells and purified protein had a marked pink colouration, although over a period of several weeks the protein became yellow, presumably owing to slow oxidation of the cofactor.

## 3. Crystallization   

**Figure 3:**
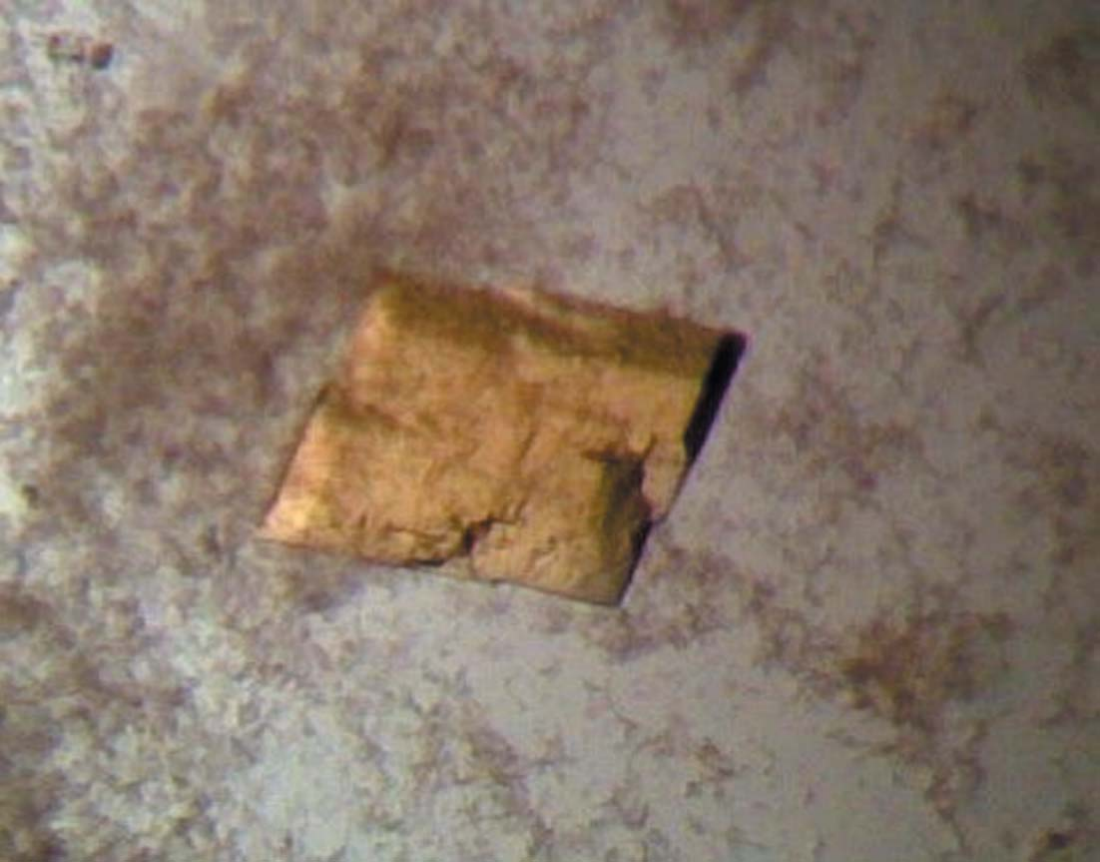
Crystals of *B. megaterium* PBGD obtained by the hanging-drop method. Their marked yellow colouration probably arises from oxidation of the dipyrromethane cofactor. The rather irregular crystal shown here is approximately 0.3 mm in its longest dimension and was separated into smaller pieces for data collection.

The purified native PBGD was concentrated to 2.5 mg ml^−1^ using a Vivaspin centrifugal concentrator and was subjected to screening for crystallization conditions by use of the hanging-drop method with Molecular Dimensions Structure Screens I and II. After about two weeks, yellow crystals appeared in Structure Screen I condition 15 (0.1 *M* sodium cacodylate pH 6.5, 0.2 *M* magnesium acetate, 20% PEG 8K) at room temperature (Fig. 3 ▶). Subsequent optimization screens revealed that crystals could be obtained reproducibly in 0.1 *M* sodium cacodylate pH 6.5–6.8, 0.2 *M* magnesium acetate, 25–30% PEG 8K. Removal of the His tag (as described above) was found to be necessary to obtain crystals of this enzyme. Selected crystals were treated by the addition of glycerol to approximately 40%(*v*/*v*) before mounting in loops and flash-cooling with an Oxford Cryosystems cryocooler.

## 4. Preliminary X-ray analysis   

**Figure 4:**
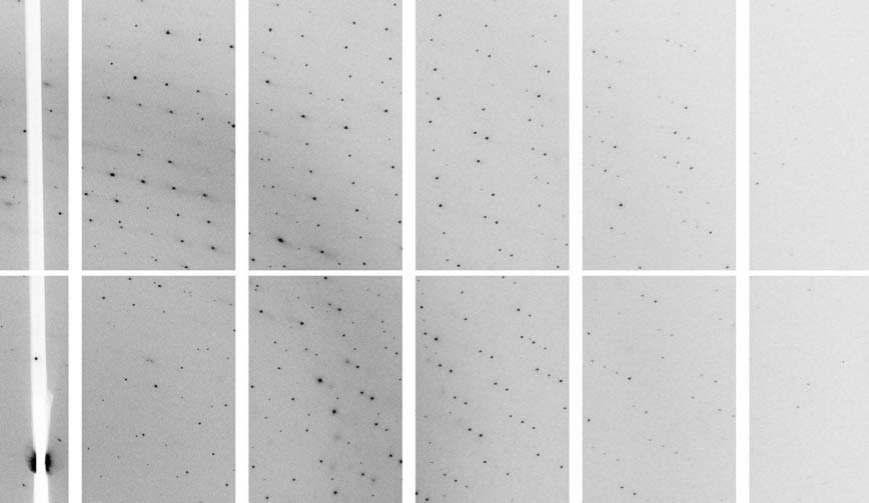
A diffraction image of *B. megaterium* PBGD obtained on DLS beamline I03 using a PILATUS 6M-F detector. The diffraction data obtained from this crystal were processed to a resolution of 1.46 Å.

X-ray data collection using station I03 at the Diamond Light Source (DLS, Didcot, England) revealed that the *B. megaterium* PBGD crystals were of very high diffraction quality (Fig. 4 ▶). Using 1° oscillations, 190° of data were collected from a single crystal maintained at a temperature of 100 K using a PILATUS 6M-F detector with an exposure time of 1 s per image (15% transmission) and a crystal-to-detector distance of 268.8 mm. The incident beam had a wavelength of 0.976 Å. Data processing with *MOSFLM* (Leslie, 2006 ▶), *SCALA* (Evans, 2006 ▶) and other programs in the *CCP*4 suite (Winn *et al.*, 2011 ▶) revealed that the crystals belonged to the monoclinic space group *P*2_1_2_1_2_1_, with unit-cell parameters *a* = 53.3, *b* = 65.8, *c* = 97.2 Å. Inspection of the correlation coefficient for half-data-set intensities, as recommended by Karplus & Diederichs (2012 ▶) and Evans (2012 ▶), suggests that the diffraction data extend to a resolution of *d*_min_ = 1.46 Å with an overall *R*_merge_ of 6.1% and an *R*_meas_ of 6.7% (for details, see Table 1 ▶). By using the method of Matthews (1968 ▶), as implemented by Kantardjieff & Rupp (2003 ▶), it was estimated that the crystals contained a single PBGD monomer per crystallographic asymmetric unit with a solvent content of 53%. Structure analysis by use of the molecular-replacement program *MOLREP* (Vagin & Teplyakov, 2010 ▶) with *E. coli* PBGD (47% identity; PDB entry 1pda; Louie *et al.*, 1992 ▶) as the search model was successful and refinement of the *B. megaterium* PBGD structure is currently in progress using this high-resolution data set.

## Figures and Tables

**Table 1:** Data-collection and processing statistics for *B. megaterium* PBGD Values in parentheses are for the outer resolution shell.

Beamline	I03, DLS
Wavelength (Å)	0.976
Space group	*P*2_1_2_1_2_1_
Unit-cell parameters	
*a* (Å)	53.3
*b* (Å)	65.8
*c* (Å)	97.2
Mosaic spread (°)	0.26
Resolution (Å)	48.60–1.46 (1.53–1.46)
*R*_merge_ † (%)	6.1 (55.9)
*R*_meas_ ‡ (%)	6.7 (61.3)
Completeness (%)	100.0 (100.0)
Average *I*/σ(*I*)	14.4 (3.0)
Multiplicity	6.2 (6.0)
No. of observed reflections	378329 (52575)
No. of unique reflections	60772 (8748)
Wilson plot *B* factor (Å^2^)	15.8
Solvent content (%)	53.0
No. of molecules per asymmetric unit	1

† *R*_merge_ = 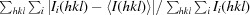.

‡ *R*_meas_ = 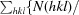 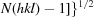 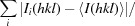 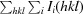, where 〈*I*(*hkl*)〉 is the mean intensity of the *N*(*hkl*) observations *I*_*i*_(*hkl*) of each unique reflection *hkl* after scaling.
